# The environmental impact of intravenous antimicrobial therapies: a comparison of OPAT and inpatient administration care pathways

**DOI:** 10.1093/jacamr/dlaf030

**Published:** 2025-03-11

**Authors:** Ann Cole, Julie Aspin, Steven Laird, Flavio Acri, Saori Galley, Michael Collins

**Affiliations:** Government Affairs and Market Access, Baxter Healthcare Limited, Compton, UK; EHS and Sustainability, Baxter Healthcare Limited, Compton, UK; Microbiology, University Hospitals Coventry and Warwickshire (UHCW) NHS Trust, Coventry, UK; Marketing, Baxter Healthcare Limited, Compton, UK; Consulting, Environmental Resources Management (ERM), London, UK; Consulting, Environmental Resources Management (ERM), London, UK

## Abstract

**Objectives:**

The urgent global threats of the climate crisis and antimicrobial resistance have the potential to be addressed in part by increasing the use of outpatient antimicrobial therapy (OPAT). Our study aimed to appraise the environmental impact of three commonly used OPAT pathways, and the traditional inpatient model of IV antimicrobial therapy.

**Methods:**

We assessed the CO_2_, waste and water footprint associated with self-care, nurse assisted and outpatient OPAT care pathways and inpatient administration of intravenous antibiotics to adult patients for whom OPAT was a viable treatment option.

**Results:**

We found that the administration of IV antibiotics in hospital is associated with a substantial environmental impact compared to OPAT. When OPAT is self-administered in the patient’s home, we discovered a CO_2_ reduction of 85%, a water use reduction of 78% and a 91% reduction in the amount of waste generated compared with the traditional inpatient treatment pathway. Nurse administered OPAT, either in the home or outpatient department, also results in lower use of water, generation of waste and CO_2_ emissions compared to the inpatient pathway.

**Conclusions:**

Our study demonstrates that using OPAT reduces the environmental impact of delivering antimicrobial therapy compared to inpatient treatment. As it is delivered away from the hospital setting, it may also reduce the risks to health associated with inpatient care. While we have shown that the reduction in CO_2_ emissions, water used and waste generated is significant for all three forms of OPAT studied, the greatest impact is seen with the use of self-administration OPAT.

## Introduction

The urgent global threats of the climate crisis and antimicrobial resistance (AMR) cannot be overstated and have long been identified as two of the world’s top health emergencies. The complex parallels between them need to be addressed immediately and from a One Health perspective, defined as an integrated, unifying approach that aims to sustainably balance and optimize the health of people, animals and ecosystems.^1^ A One Health approach recognizes that the health of humans, domestic and wild animals, plants and the wider environment (including ecosystems) are closely linked and inter-dependent.

The impact of the climate crisis on health is likely to be catastrophic, with increasing AMR, food insecurity, air pollution, pollen and food safety risks, changing exposure to heat and cold, disrupted access and functioning of health services and facilities, flooding, wild fires, clean water shortages, water-borne diseases and increased exposure to ultra violet radiation.^2^ Healthcare’s climate footprint is 4.4% of the global total; meaning if it were a country, it would be the fifth largest emitter on the planet.^3^ Moreover, hospital buildings and their activities have an excessive impact on the climate. They release greenhouse gases through transport, the use and disposal of products, and consume more electricity and energy per square metre than any other non-residential building.^4–6^ As Keller *et al*. described in their analysis in 2021, ‘hospital building infrastructure and catering are the main contributors for various environmental impacts, followed by heating and electricity. Waste and wastewater, pharmaceuticals, and medical and housekeeping products are also relevant.’^7^

The recent independent investigation of the National Health Service (NHS) in England by Lord Darzi^8^ states that too much focus has been placed on care in hospitals, yet the advantages of care closer to home are clear. Treating patients in the community reduces the burden on an already overstretched NHS secondary care service, potentially reduces the environmental impact of treatment and can lower the risk of transmission of drug-resistant microorganisms.

It has been predicted that AMR will be responsible for 10 million deaths per year globally by 2050.^9^ While the overuse of antimicrobials is undoubtedly one of the main drivers of AMR, reportedly growing 65% between 2010 and 2015,^10^ and exacerbated by the COVID-19 pandemic,^1^ the ability to effectively treat infections is a vital component of modern healthcare. Nevertheless, resistant organisms are often more expensive to treat than non-resistant infections and can increase the length of hospital stay by up to 9 days.^11^ A clinically appropriate and environmentally responsible approach to the use of antimicrobial therapy is therefore vital.

Reducing hospital activity offers huge opportunities. Treating patients at home can minimize the risk of hospital acquired infections, which are often resistant to antimicrobials due to their high usage in the hospital setting.^1,12^ By reducing the need for prolonged hospital stays, and where possible avoiding hospital admissions altogether, patients are exposed to fewer antibiotics, which diminishes the selective pressure that leads to the emergence of resistant organisms.^13^ Indeed, out-of-hospital care is a strong theme in the ‘Delivering a net zero NHS’ strategy, as it supports a reduction in carbon emissions, waste, water use, travel, freight and manufacturing.^14^

While strategies such as decreasing antimicrobial therapy duration and promoting stepdown to oral agents are important, outpatient parenteral antimicrobial therapy (OPAT) allows patients who need intravenous (IV) antimicrobial therapy and who are well enough to receive care away from the hospital setting to be treated in the community. OPAT is frequently used as a vehicle to deliver narrow spectrum antibiotics and is often cited as a strategy to reduce AMR, for example in the UK Government antimicrobial toolkit,^15^ and the UK 5-year action plan for antimicrobial resistance 2024 to 2029.^16^ It is a cost effective^17^ and clinically viable alternative to inpatient treatment^18,19^; however, to date and to the best of our knowledge, there have been no studies that assess the environmental impact of the different OPAT care models in comparison to hospital-based delivery of IV antimicrobials.

Our study aimed to appraise the carbon emissions [greenhouse gas emissions (GHG) to air, contributing to climate change, expressed as caron dioxide (CO_2_) equivalent], waste and water footprint implications associated with IV antimicrobial therapy. We explored the environmental impact of three commonly used OPAT pathways, and the traditional inpatient model of IV antimicrobial therapy.

## Methods

We assessed the environmental impact of three OPAT care pathways and inpatient administration of IV antibiotics to adult patients (>18 years of age) in the NHS in England for which OPAT was a viable treatment option.^20^ The unit of analysis was 10 adult patients, based on the available capacity for patients on the regional NHS (publicly funded) OPAT service offered at any one time by a leading service provider.^21^ Ceftriaxone was chosen due to its common use throughout the UK as part of OPAT services. The calculations used to represent duration of treatment in the modelling for each care pathway were based on those used by Dimitrova *et al.* using a weighted average of 15.3 days.^17^ The four care pathway scenarios were:

Scenario 1: Self-administration OPAT. the patient is provided with medication, equipment and training to facilitate daily self-administration of the course of antibiotics using an elastomeric pump device in their own home.

Scenario 2: Nurse-assisted OPAT administration. A nurse visits the patient every day to administer the course of antibiotics using an elastomeric pump device in their own home.

Scenario 3: Hospital OPAT administration. The patient travels to the hospital outpatient department daily to receive the course of antibiotics using an elastomeric pump device.

Scenario 4: Inpatient administration. The patient receives the course of antibiotics as a hospital inpatient via IV bolus.

All known activities were included in the scope of each care pathway scenario (Table 1) and are based on the services routinely provided in the UK by the NHS and Baxter Healthcare Limited.

**Table 1.** Description of antimicrobial therapy care pathway scenarios and study assumptions^17,22^

Activity	Scenario 1: self-care OPAT	Scenario 2: nurse administration OPAT	Scenario 3: hospital OPAT	Scenario 4: hospital inpatient IV antimicrobial
Intravenous access insertion	PICC or midline catheter. The insertion procedure is performed by a trained nurse in a treatment room and takes ∼1 hour.			IV cannula. The procedure takes ∼10 minutes
Patient training	1-hour home training session by one nurse. Guidance literature is also provided	Not required	Not required	Not required
Elastomeric pump device manufacture	One elastomeric device used per dose delivered			Not required
Drug Reconstitution	Ceftriaxone 1 g reconstituted in 10 mL of saline, and diluted in 90 mL of saline			Ceftriaxone 1 g reconstituted in 10 mL saline
Patient travel from hospital to home	Not accounted for in the OPAT pathway			Required at end of treatment
Nurse travel to patient’s home	Not applicable	Daily, to administer OPAT	Not applicable	Not applicable
Patient travel to hospital	Not applicable	Not applicable	Daily, for outpatient treatment	Not applicable
Patient travel to hospital for blood tests and prescription	Weekly during OPAT treatment	Not applicable	Not applicable	Not applicable
Drug administration	IV ceftriaxone 1 g per 100 mL o/d for ∼15.3 days. One elastomeric device per dose administered	IV ceftriaxone 1 g per 100 mL o/d for ∼15.3 days. One elastomeric device used per dose administered at home by the nurse	IV ceftriaxone 1 g per 100 mL o/d for ∼15.3 days. One elastomeric device used per dose administered by a nurse at the hospital outpatient department, assuming 1 hour of nurse time	IV ceftriaxone 1 g per 100 mL o/d for ∼15.3 days administered via IV bolus.
Inpatient stay	Not applicable	Not applicable	Not applicable	∼22.5 bed days assuming low intensity, general ward
Blood test	Weekly blood test to monitor effectiveness of treatment			
Patient assessment	End of treatment assessment involves four clinicians for 1 hour per 10 patients			
Patient travel to hospital if OPAT unsuccessful	Assumed in 6.4% of the patient population			Not applicable
Inpatient stay if OPAT unsuccessful	∼11.25 inpatient bed days (low intensity) required, assumed in 6.4% of the patient population)			Not applicable

The method for undertaking a care pathway assessment is described in *Care Pathways: Guidance on Appraising Sustainability*.^23^ This begins with the scoping stage, which defines the objectives and unit of analysis, along with mapping of the care pathway activities and undertaking a materiality assessment. A materiality assessment is a high-level study that explores the estimated and relative impact of the study’s components to prioritize data collection efforts. An environmental emission factor is assigned to each activity included in the care pathway to calculate the environmental impact. The materiality assessment identifies where more detailed data collection is needed, to generate a more focused environmental impact assessment of the care pathway. Following the materiality assessment, additional data collection are completed where it has been identified there is a need, and the environmental impact calculations are updated.

The scoping stage of this study was carried out by the authors using expert opinion along with relevant source documents.^17,22,23^ All the activities associated with the treatment of patients with IV antibiotics were mapped and their likely impact was estimated to inform data collection and areas of uncertainty. The data collection and assessment stage improved the estimate of GHG emissions (CO_2_), water use, and waste generated for each of the four antimicrobial therapy scenarios by using data from the following sources:

- Sustainable Healthcare Coalition Care Pathways Guidance^23^ for life cycle-based emission factors of specific healthcare activities, the emission factors include GHG contributions from staff travel, energy use, waste supply and disposal, equipment and consumable manufacture and disposal.
- ecoinvent (https://ecoinvent.org/) for specific emission factors of consumable materials, where they were known and available.
- The Association of the British Pharmaceutical industry Blister Pack Carbon Evaluation Tool for emission factors of Active Pharmaceutical Ingredients (API) and their transport, formulation and packaging. (Generic drug substance environmental intensity data was used to characterize the production of antibiotics).
- Patient data assumptions for the different antimicrobial treatment options, relating to factors such as length of treatment and numbers of OPAT patients requiring re-hospitalization, were also incorporated into the calculations based on the Supplementary data used by Dimitrova *et al.* in their health economic assessment.^17^ These assumptions include:
  - A nurse travels on average 11 miles per journey to patient’s home.
  - Consumables used for each administrations patient include one apron, one pair of gloves, four needles, four syringes, one pre-injection swab and three 0.9% sodium chloride ampoules.
  - OPAT patients would have a peripherally inserted central catheter (PICC line) whereas inpatients would have an IV cannula.
- A streamlined life cycle assessment was undertaken to appraise the environmental impact of one commonly used elastomeric pump device. The elastomeric pump device is required for administering the antimicrobial therapy for the three OPAT scenarios. The system boundary of the life cycle assessment was cradle to grave, accounting for all inputs and outputs associated with the life cycle of the device: raw material sourcing, device manufacture and assembly, sterilization, transportation by sea and road to the UK, use and disposal by hazardous waste incineration without energy recovery.^20^ Activity data were collected to represent each stage of the life cycle of the elastomeric pump (e.g. quantities of raw material, electricity consumptions, transportation distances), which were then multiplied by carbon, water and waste characterization factors, representing effects per unit of activity (e.g. kg CO_2_ equivalents per kg of polypropylene, m^3^ water per kg of polypropylene, kg waste per kg of polypropylene). The effects of all the activities were then summed together to determine the environmental impact of the elastomeric pump.

## Results

The environmental effects (CO_2_ emissions, waste generated and water usage) for each care pathway are shown for the patient population (10 patients) of each care pathway in Table 2 and summarized in Table 3.

**Table 2.** Environmental impact per patient population (10 patients)

	Self-care OPAT			Nurse-assisted OPAT			Outpatient OPAT			Inpatient IV administration		
Stage	Climate kgCO_2_ eq (%)	Water m^3^ (%)	Waste kg (%)	Climate kgCO_2_ eq (%)	Water m^3^ (%)	Waste kg (%)	Climate kgCO_2_ eq (%)	Water m^3^ (%)	Waste kg (%)	Climate kgCO_2_ eq (%)	Water m^3^ (%)	Waste kg (%)
Vascular access insertion	37.8 (4)	47.8 (2)	5.08 (9)	37.8 (3)	47.8 (2)	5.08 (9)	37.8 (2)	47.8 (2)	5.08 (6)	0.81 (0)	0.40 (0)	0.20 (0)
Patient training	5.51 (1)	23.7 (1)	0.688 (1)	n/a	n/a	n/a	n/a	n/a	n/a	n/a	n/a	n/a
Device manufacture	139 (15)	1551 (65)	11.9 (22)	139 (11)	1551 (64)	11.9 (22)	139 (8)	1551 (53)	15 (19)	n/a	n/a	n/a
Drug reconstitution	292 (30)	412 (17)	5.13 (9)	292 (23)	412 (17)	5.13 (10)	292 (16)	412 (14)	5.13 (6)	93.5(1)	337 (3)	0.02 (0)
Drug administration	181 (18)	52.5 (2)	14.4 (27)	181(14)	52 (2)	14.4 (27)	292 (16)	448 (15)	35.4 (46)	368 (6)	943 (9)	71.3 (12)
OPAT Helpline	0.114 (0)	0.92 (0)	n/a	n/a	n/a	n/a	n/a	n/a	n/a	n/a	n/a	n/a
Nurse travel to patient home	n/a	n/a	n/a	433 (34)	49.0 (2)	—	n/a	n/a	n/a	n/a	n/a	n/a
Blood tests	2.53 (0)	0.022 (0)	1.26 (2)	2.53 (0)	0.022 (0)	1.26 (2)	2.53 (0)	0.0218 (0)	1.26 (2)	2.53 (0)	0.022 (0)	1.26 (0)
Patient travel for blood tests and prescriptions	126 (13)	23.1 (1)	n/a	n/a	n/a	n/a	n/a	n/a	n/a	n/a	n/a	n/a
Patient travel to outpatient dept	n/a	n/a	n/a	n/a	n/a	n/a	885 (48)	162 (6)	—	n/a	n/a	n/a
Patient assessment	0.09 (0)	0.2 (0)	—	0.092 (0)	0.24 (0)	—	0.092 (0)	0.24 (0)	—	0.092 (0)	0.24 (0)	—
Inpatient stay	n/a	n/a	n/a	n/a	n/a	n/a	n/a	n/a		5782 (90)	9276 (85)	503 (85)
Patient travel to re-hospitalization	3.71 (0)	0.68 (0)	—	3.71 (0)	0.68 (0)	—	3.71 (0)	0.68 (0)	—	n/a	n/a	n/a
Re-hospitalization	185 (19)	297 (12)	16.1 (30)	185 (14)	297 (12)	16.1 (30)	185 (10)	297 (10)	16.1 (21)	185 (3)	297 (3)	16.1 (3)
Total	972 (100)	2429 (100)	57.2 (100)	1284 (100)	2430 (100)	56.5 (100)	1847 (100)	2939 (100)	77.5 (100)	6433 (100)	10 853 (100)	592 (100)

**Table 3.** Environmental impact per patient population

	Impact per patient population		
Stage	Climate impact kg CO_2_ eq	Water impact m^3^	Waste impact kg
Scenario 1: self-administration OPAT	972	2409	54.5
Scenario 2: nurse-assisted administration OPAT	1273	2410	53.8
Scenario 3: hospital outpatient administration OPAT	1837	2918	74.8
Scenario 4: inpatient administration IV antibiotics	6433	10 853	592

### Scenario 1: self-administration OPAT

The most significant contributor towards the environmental impact of self-administration OPAT is the manufacture of the device that accounts for 65% of the water used in this pathway, 22% of the total waste produced and 15% of CO_2_ emissions. The reconstitution and administration of the drug also make a considerable contribution, accounting for 30% of the CO_2_ emissions, 17% of water used and 9% of the waste. Re-hospitalization, which is required for only ∼6.4% of patients if the infection is not successfully treated,^17,24^ contributes 30% of the waste produced, 19% of CO_2_ emissions and 12% of all the water used in this care pathway.

### Scenario 2: nurse-assisted OPAT administration

As with self-administration OPAT, there is a significant environmental impact from the re-hospitalization of the minority of patients whose infection is not successfully treated. Similarly, the device manufacture and drug administration also make considerable contributions to CO_2_ emissions, water use and waste produced. However, in this scenario, the nurse travels to the patients home each day to administer the antimicrobial therapy and this contributes 433 kg CO_2_ eq that accounts for more than one-third (34%) of the total climate impact within the pathway.

### Scenario 3: hospital OPAT administration

When patients travel to the outpatient department to receive OPAT, the relative contribution of water used in the manufacture of the device reduces from 65% (in the home-based OPAT scenarios) to 53%. A larger contribution to the water use, compared to the two other OPAT pathways, is from the drug administration which increases from ∼52 to 448 m^3^. Drug administration also makes a considerable contribution towards the waste produced (46%) and accounts for 16% of CO_2_ emissions. The highest contribution of GHG emissions comes from the patient’s travel to the outpatient department each day, which is 885 kg CO_2_ eq and makes up almost half (48%) of the carbon emissions in this scenario.

### Scenario 4: inpatient administration

The low-intensity bed stay required for inpatient administration of IV antibiotics is the most significant contributor to all three modules (GHG emissions, water and waste), accounting for over 85% of each of the environmental effects assessed. This is driven by the consumables (including food) and energy associated with a patient staying in a hospital.^25^ As with the outpatient OPAT pathway, drug administration also makes a significant contribution towards the environmental impact of inpatient IV antibiotic therapy (generating 368 kg CO_2_ eq, using 943 m^3^ water and producing >71.3 kg of waste) due mainly to the clinician’s time and consumables used.

## Discussion

IV antimicrobial therapy is an essential and often lifesaving treatment, but it has a high environmental impact, adversely affecting the global climate and exacerbating the risks of AMR.^1,26^ Our study, which assessed the environmental footprint of three different OPAT scenarios and the traditional inpatient model, found that the administration of IV antibiotics in hospital is associated with a substantial environmental impact compared to OPAT.

When OPAT is self-administered at the patient’s home, we found a CO_2_ reduction of 85%, a water use reduction of 78% and a 91% reduction in the amount of waste generated compared with the traditional inpatient treatment pathway. This equates to a reduction in CO_2_ of 5461 kg CO_2_ eq per patient population, or emissions from driving a petrol-fuelled passenger car 13 907 miles (the equivalent of driving from London to Rome and back six times). When nurses administer OPAT in the patient’s home, it results in a CO_2_ reduction of 80%, water reduction of 78% and waste reduction of 91%, compared with the inpatient administration of IV antimicrobial therapy. This equates to a reduction in CO_2_ of 5160 kg CO_2_ eq per patient population, or emissions from driving or emissions from driving a petrol-fuelled passenger car 13 140 miles. Administering OPAT to patients in the outpatient department also represents a reduction of the environmental impact compared with the traditional inpatient treatment pathway resulting in a 71% reduction of CO_2_ generated, a 73% reduction of water used and an 87% waste reduction. This equates to a reduction in CO_2_ of 4596 kg CO_2_ eq per patient population, or emissions from driving, or emissions from driving a petrol-fuelled passenger car 11 740 miles.

Our analysis identified that the most substantial contributions to the environmental impact of the antimicrobial therapy care pathway are travel (both for patients and nurses), and inpatient low-intensity bed stay to receive IV antibiotics. Inpatient care is primarily associated with the use of energy and consumables and is therefore directly related to the length of hospital stay. The environmental impact of patient and nurse travel is primarily associated with passenger vehicle transport.

The elastomeric pump used in all three OPAT treatment pathways contributes between 8% and 15% of the CO_2_ produced, 53%–65% of the water used and 19%–22% of the waste generated. However, the overall environmental impact of OPAT is substantially lower than that from the inpatient IV antimicrobial treatment pathway.

OPAT is a more responsible, clinically effective and cost-effective method of delivering antimicrobial therapy outside the hospital for appropriate patients who are otherwise medically fit.^17,18,24,27–29^ Our study demonstrates that using OPAT reduces the environmental impact of delivering antimicrobial therapy compared to inpatient treatment, and as it is delivered away from the hospital setting it may also reduce the risks to health associated with inpatient care. While we have demonstrated that the reduction in CO_2_ emissions, water used and waste generated is significant for all three forms of OPAT studied, the greatest impact is seen with the use of self-administration OPAT. Moreover, Bodycot *et al*.^19^ found that up to 75% of patients receiving IV antimicrobials are suitable for self-administration of OPAT.

Our study should be interpreted bearing in mind some limitations related to data quality and the use of secondary data and assumptions for some processes and activities, including the following: the use of average environmental intensity data from the Sustainable Healthcare Coalition care pathway guidance for common modules,^23,30^ the use of generic drug substance environmental intensity data to characterize production of antibiotics and use of secondary life cycle emission factor data to characterize the impact of manufacture of consumables. The care pathway assessment does not include visitor travel, patient travel associated with the original treatment from which infection occurred or the original treatment itself.

We acknowledge that there is scope to improve the accuracy of the assessment, focussing on: replacing the GHG emission estimates from literatures with specific environmental information relating to the manufacture and supply of antibiotics and consumables; patient travel, as the distance to outpatient departments and inpatient settings and specific audits of material, energy and staff requirements associated with patients being treated for infections in hospital with antimicrobial therapy.

Future studies could involve investigations to better understand the scalability of the positive environmental impact of OPAT, particularly in the real-world setting. We also acknowledge that there are many other comparisons that can be studied, for example comparing the environmental impact of oral antibiotics with OPAT, and comparing OPAT pathways using different antimicrobial agents, oral and combination therapies.
